# Dual use of electronic and conventional cigarettes is associated with higher cardiovascular risk factors in Korean men

**DOI:** 10.1038/s41598-020-62545-3

**Published:** 2020-03-27

**Authors:** Choon-Young Kim, Yu-Jin Paek, Hong Gwan Seo, Yoo Seock Cheong, Cheol Min Lee, Sang Min Park, Da Won Park, Kiheon Lee

**Affiliations:** 1 Department of Family Medicine, Seoul National University Bundang Hospital, Seongnam, Korea; 2 Department of Family Medicine, Health Promotion Center, Hallym University Sacred Heart Hospital, Anyang, Korea; 3 Center for Cancer Prevention and Detection, National Cancer Center, Goyang, Korea; 4 Department of Cancer Control and Population Health, National Cancer Center Graduate School of Cancer Science and Policy, Goyang, Korea; 5 Department of Family Medicine, Dankook University College of Medicine, Cheonan, Korea; 6 Department of Family Medicine, Healthcare System Gangnam Center, Seoul National University Hospital, Seoul, Korea; 7 Department of Family Medicine, Seoul National University Hospital, Seoul, Korea; 8 Department of Family Medicine, Seoul National University College of Medicine, Seoul, Korea; 9 Department of Biomedical Sciences, Seoul National University Graduate School, Seoul, Korea

**Keywords:** Addiction, Metabolic syndrome, Risk factors

## Abstract

Most smokers who use electronic cigarettes (e-cigarettes) to stop smoking simultaneously use conventional cigarettes (dual users). We aimed to compare the prevalence of cardiovascular risk factors among dual users, cigarette-only smokers, and never smokers in Korean men. We used data acquired from Korean National Health and Nutrition Examination Survey (2013–2017) pertaining to 7,505 male participants aged 19 years or older. About 85% of e-cigarette users were dual users. Dual users had greater nicotine dependence and higher urinary cotinine levels than cigarette-only smokers. Dual users had more psychosocial and behavioural risk factors, including perceived high stress, depressive mood, high daily intake of energy, and obesity, than never smokers and cigarette-only smokers. The prevalence of metabolic syndrome (MetS) was higher among dual users, and their multivariate-adjusted prevalence odds ratio for MetS was 2.79 (P < 0.001) compared with never smokers and 1.57 (P = 0.038) compared with cigarette-only smokers. Given that most e-cigarette users are dual users and dual users are more vulnerable to cardiovascular risk factors than cigarette-only smokers and never smokers, more active treatment for smoking cessation and intensive lifestyle interventions for dual users should be considered with priority.

## Introduction

The smoking rate in Korean men is the fourth highest among OECD countries^1^, primarily because of the low price of tobacco products. After the Korean government increased the price of cigarettes by 80% in 2015 as part of intensive efforts to reduce tobacco use, the overall smoking rate declined from 66.3% in 1998 to 39.4% in 2015, a decreasing trend that continues to the present day. However, use of the electronic cigarette (e-cigarette), introduced in Korea in 2007, has rapidly increased from 2.0% in 2013 to 7.1% in 2015, according to the Korean National Health and Nutrition Examination Survey (KNHANES) data^2^. E-cigarettes have been marketed as healthier alternatives to cigarettes or as smoking cessation aids^3^. However, most e-cigarette users do not stop smoking and instead use both e-cigarettes and cigarettes (dual users). Recent studies have shown that dual users are associated with a higher risk of cardiovascular disease (CVD) than cigarette-only smokers^4–6^. In addition to harmful substances in cigarettes and e-cigarettes, several modifiable psychosocial and behavioural risk factors are associated with an increased risk of CVD^7,8^. Metabolic syndrome (MetS) is a cluster of cardiovascular risk factors and characterized by abdominal obesity, atherogenic hyperlipidaemia (elevated triglycerides or reduced high-density lipoprotein [HDL]-cholesterol), high blood pressure (BP), and elevated fasting glucose levels^9^. Psychosocial and behavioural risk factors, such as stress/depressive mood, obesity, decreased physical activity, and high fat and alcohol consumption, among smokers are all closely connected to and overlap with cardiovascular risk factors^9^. Accumulated weight gain in smokers who failed to quit smoking many times due to high nicotine dependence may worsen the cardiovascular risk factors^10^. It is important for health professionals to have a better understanding of modifiable cardiovascular risk factors among dual users, in addition to providing more active treatments for smoking cessation and intensive lifestyle interventions. However, currently the data available to assess the effects of cardiovascular risk factors among dual users are either insufficient or fragmented. Therefore, in this study, we aimed to compare the prevalence of cardiovascular risk factors among dual users, cigarette-only smokers, and never smokers in Korean men.

## Results

We categorized the 7,505 participants into three groups: dual users (n = 337, 5.1%), cigarette-only smokers (n = 4,079, 53.4%), e-cigarette-only users (n = 62, 0.9%), and never smokers (n = 3,027, 40.7%). Most of the e-cigarette users were dual users (85.3% (n = 337) for current smokers, 13.1% (n = 53) for former smokers, and 1.6% (n = 9) for never smokers).

### Socioeconomic, psychosocial and behavioural risk factors

The mean age was 36.7 years for dual users, and it was significantly lower than that of cigarette-only smokers and never smokers (P < 0.001). The proportion of dual users was the highest among those aged 19–29. The proportions of highly educated and high-income participants, those living in urban areas, those living alone (including never-married, separated, widowed, and divorced men), and professional workers were significantly higher in dual users than in cigarette-only smokers (all P < 0.05). Dual users had significantly higher proportions of perceived high stress and continuous depressive mood lasting for more than 2 weeks than cigarette-only smokers and never smokers (all P < 0.001). Dual users perceived their health status as worse and had higher proportion of high-risk drinking compared to never smokers (all P < 0.001). Dual users ingested more calories per day and had higher proportion of all classes of obesity than cigarette-only smokers (P = 0.006 and P = 0.014) and never smokers (P < 0.001 and P = 0.007) (Table 1).

**Table 1:** Socioeconomic status, psychosocial and behavioural risk factors by smoking status.

	Dual users	cigarette-only smokers^a^	Never smokers^b^
	(n = 337)	(n = 4,079)	(n = 3,027)
**Age (years)**	36.7 ± 0.7	43.6 ± 0.3**	39.8 ± 0.4**
19–29	87 (32.9)	496 (17.7)**	761 (35.9)**
30–39	100 (30.5)	859 (23.8)**	564 (20.9)**
40–49	78 (22.2)	943 (25.4)**	466 (15.4)**
≥50	72 (14.5)	1781 (33.1)**	1236 (27.8)**
**Educational level**			
<High school	41 (10.9)	919 (17.7)*	551 (12.2)
≥High school	271 (89.1)	2947 (82.3)*	2353 (87.8)
**Household income**			
1^st^–2^nd^ quartile (low)	159 (45.9)	2204 (54.4)*	1430 (48.5)
3^rd^–4^th^ quartile (high)	178 (54.1)	1855 (45.6)*	1581 (51.5)
**Residence location**			
Rural	115 (33.3)	1744 (41.3)*	1156 (37.6)
Urban	222 (66.7)	2335 (58.7)*	1871 (62.4)
**Marital status**			
Married with spouse present	191 (48.4)	2856 (65.3)*	1930 (53.6)
Single	146 (51.6)	1218 (34.7)*	1096 (46.4)
**Occupational status**			
Manual labour or unemployed	193 (61.9)	2819 (71.1)*	1933 (65.1)
Professional jobs	119 (38.1)	1031 (28.9)*	965 (34.9)
**Mental illness**			
Perceived high stress	138 (41.9)	1196 (30.7)**	639 (23.0)**
Depressive mood	43 (20.9)	229 (8.9)**	147 (7.6)**
Suicidal thoughts	16 (7.1)	126 (4.9)	53 (2.3)*
**Poor self-rated health status**	231 (73.3)	2838 (72.0)	1690 (55.5)**
**Decreased physical activity**	131 (44.8)	1597 (50.2)	1000 (39.0)
**High-risk drinking**	100 (29.8)	1135 (29.7)	280 (9.1)**
**Daily energy intake (1,000** **kcal/d)**	2.76 ± 0.10	2.48 ± 0.02*	2.39 ± 0.02**
**Body mass index (kg/m**^2^**)**			
Normal (18.5–22.9)	84 (26.1)	1341 (35.1)*	998 (35.5)*
Pre-obese (23–24.9)	78 (22.9)	944 (23.8)*	732 (25.3)*
Obese class I-III (≥25)	160 (51.0)	1555 (41.2)*	1119 (39.2)*
**Comorbidity**			
Hypertension	31 (5.3)	693 (12.9)*	518 (11.3)*
Diabetes	17 (3.3)	341 (5.9)*	199 (4.4)
Hyperlipidaemia	16 (4.2)	275 (5.3)	175 (4.0)
Cardiovascular disease	10 (1.8)	181 (2.9)	108 (2.1)
**Family history**			
Hypertension	133 (38.9)	1288 (33.2)	948 (32.4)*
Diabetes	70 (22.2)	777 (20.0)	439 (15.4)*
Hyperlipidaemia	22 (8.4)	158 (4.5)*	157 (5.7)
Cardiovascular disease	49 (14.2)	622 (14.9)	356 (9.8)*
**Urinary cotinine (µg/mL)**^**c**^	1303.4	1236.1	0.7
	(850.2–1925.0)	(677.7–1800.0)*	(0.4–1.4)**

Results are expressed as the mean ± standard error or unweighted numbers (weighted %). P-values are calculated using complex samples general linear model or chi-square tests (*P < 0.05, **P < 0.001). ^a^P-values are calculated between dual users and cigarette-only smokers. ^b^P-values are calculated between dual users and never smokers. ^c^Urinary cotinine level is expressed as median value (interquartile range) because it does not follow a normal distribution.

### Smoking behaviours

The three primary reasons given by current e-cigarette users for using e-cigarettes were that they helped with smoking cessation (45.9% for dual users, 55.7% for e-cigarette-only users), they are less harmful than conventional cigarettes (20.2% for dual users, 18.8% for e-cigarette-only users), and they produce less smell than conventional cigarettes (17.4% for dual users, 13.7% for e-cigarette-only users). The number of cigarettes smoked per day (CPD) was not significantly different between dual users and cigarette-only smokers (15.1 cigarettes/day vs. 14.8 cigarettes/day, P = 0.589). The mean age of cigarette smoking initiation was significantly lower in dual users than in cigarette-only smokers (18.2 years vs. 19.0 years, P = 0.001), and the mean duration of cigarette smoking (18.2 years vs. 24.9 years, P < 0.001) and pack-years of cigarettes (14.4 pack-years vs. 18.6 pack-years, P < 0.001) were significantly lower in dual users than in cigarette-only smokers. Dual users had significantly higher proportion of poly-use of new tobacco products such as snus, water-pipe, or cigar than cigarette-only smokers (P < 0.001). The proportion who tried to stop smoking for more than 1 day in the past year was 67.1% for dual users and 56.4% for cigarette-only smokers (P = 0.013), and the proportion who had intentions to quit smoking (preparation or contemplation stages for smoking cessation) were 49.3% for dual users and 36.0% for cigarette-only smokers (P < 0.001). The median urinary cotinine (UC) levels were higher in dual users than cigarette-only smokers (1303.4 µg/mL vs. 1236.1 µg/mL, P = 0.011). The proportion who used nicotine replacement therapy (NRT) within 5 days was highest in e-cigarette-only users and followed by dual users and cigarette-only smokers (17.0%, 2.9%, and 0.8%, respectively; P < 0.011) (Table 2).

**Table 2:** Smoking behaviours between dual users and cigarette-only smokers.

	Dual users	Cigarette-only smokers	P-value
	(n = 337)	(n = 4,079)	
**Age of cigarette smoking initiation (years)**	18.2 ± 0.2	19.0 ± 0.1	0.001
**Frequency of cigarette smoking**			
Daily	302 (88.3)	3633 (88.6)	0.876
Occasional	35 (11.7)	446 (11.4)	
**Cigarettes smoked per day**	15.1 ± 0.4	14.8 ± 0.1	0.589
1–9 (light smoker)	47 (14.9)	771 (18.7)	
10–19 (moderate smoker)	170 (48.7)	1743 (43.5)	0.144
≥20 (heavy smoker)	120 (36.4)	1564 (37.9)	
**Duration of cigarette smoking (years)**	18.2 ± 0.8	24.9 ± 0.3	<0.001
**Pack-years of cigarettes**^**a**^	14.4 ± 0.9	18.6 ± 0.4	<0.001
**Poly-use of new tobacco products**			
Snus	6 (2.3)	21 (1.1)	
Water-pipe	6 (2.7)	6 (0.4)	<0.001
Cigar	10 (6.4)	65 (2.8)	
**Quit attempts** ≥ **1days in the past year**	116 (67.1)	1464 (56.4)	0.013
**NRT use within 5 days**	13 (2.9)	39 (0.8)	<0.001
**Preparation stage to quit smoking**			
Preparation (<1 month)	96 (29.9)	839 (20.6)	
Contemplation (<6 months)	64 (19.4)	595 (15.4)	<0.001
Pre-contemplation (no intention)	177 (50.7)	2643 (63.9)	
**Urinary cotinine (µg/mL)**^**b**^	1303.4	1236.1	0.011
	(850.2–1925.0)	(677.7–1800.0)	

Results are expressed as the mean ± standard error or unweighted numbers (weighted %). P-values are calculated using complex samples general linear model or chi-square tests. ^a^Pack-years of cigarettes was calculated as the product of the average number of packs of cigarettes smoked per day and smoking duration in years. ^b^Urinary cotinine level is expressed as median value (interquartile range) because it does not follow a normal distribution. Abbreviations: NRT, nicotine replacement therapy.

### The prevalence of metabolic syndrome and its components

The prevalence of MetS was not significantly different between dual users and cigarette-only smokers (P = 0.587), but mean waist circumference (WC) (87.4 cm vs. 85.1 cm, P < 0.001) and the prevalence of elevated WC (39.3% vs. 28.7%, P < 0.001) were significantly higher in dual users than in cigarette-only smokers. The prevalence of MetS and the number of MetS components in dual users were higher than in never smokers (all P < 0.001). Among the five components of MetS, elevated WC, elevated triglycerides, and reduced HDL-cholesterol were more prevalent in dual users than in never smokers (all P < 0.001). The prevalence of elevated BP was significantly lower in dual users than in cigarette-only smokers (P < 0.001) and never smokers (P = 0.017). The prevalence of elevated fasting glucose levels was not significantly different among three groups (Table 3).

**Table 3:** The prevalence of metabolic syndrome and its components by smoking status.

	Dual users	cigarette-only smokers^a^	Never smokers^b^
	(n = 337)	(n = 4,079)	(n = 3,027)
**Elevated WC**			
Mean WC (cm)	87.4 ± 0.6	85.1 ± 0.2**	84.2 ± 0.2**
Prevalence (%)	132 (39.3)	1205 (28.7)**	809 (25.5)**
**Elevated BP**			
Mean systolic BP (mmHg)	117.1 ± 0.7	119.1 ± 0.3*	118.2 ± 0.3
Mean diastolic BP (mmHg)	78.1 ± 0.6	78.2 ± 0.2	77.2 ± 0.2
Prevalence (%)	117 (27.3)	1779 (40.2)**	1222 (34.0)*
**Elevated fasting glucose**			
Mean fasting glucose (mg/dL)	100.9 ± 1.5	101.3 ± 0.4	97.5 ± 0.5*
Prevalence (%)	114 (33.6)	1591 (38.5)	976 (27.9)
**Elevated triglycerides**			
Mean triglycerides (mg/dL)	195.4 ± 10.6	185.9 ± 3.0	135.5 ± 2.5**
Prevalence (%)	155 (50.9)	1922 (50.0)	950 (30.8)**
**Reduced HDL-cholesterol**			
Mean HDL-cholesterol (mg/dL)	46.2 ± 0.7	46.8 ± 0.2	48.3 ± 0.2*
Prevalence (%)	120 (36.0)	1363 (32.1)	811 (24.7)**
**Number of MetS components**			
0	61 (22.7)	722 (21.7)	832 (34.8)**
1	54 (17.4)	822 (22.3)	679 (24.8)**
2	79 (27.5)	853 (21.8)	532 (17.2)**
3	57 (18.0)	721 (18.7)	427 (13.1)**
4	36 (10.8)	470 (11.3)	248 (7.0)**
5	15 (3.7)	186 (4.1)	113 (3.1)**
**Diagnosis of MetS**			
Prevalence (%)	108 (32.5)	1377 (34.2)	788 (23.2)**

Results are expressed as the mean ± standard error or unweighted numbers (weighted %). P-values are calculated using complex samples general linear model or chi-square tests (*P < 0.05, **P < 0.001). ^a^P-values are calculated between dual users and cigarette-only smokers. ^b^P-values are calculated between dual users and never smokers. Abbreviations: WC, waist circumference; BP, blood pressure; HDL, high-density lipoprotein; MetS, metabolic syndrome.

Multivariate logistic regression analyses after adjustment for variables such as age, educational level, household income, residence location, occupational status, marital status, perceived high stress, depressive mood, suicidal thoughts, self-rated health status, alcohol consumption, body mass index (BMI), comorbidities and family history of diseases (Model 3) showed that the multivariate-adjusted prevalence odds ratio (aPOR) for MetS in dual users was 2.79 (95% confidence interval [CI] = 1.72–4.53, P < 0.001) compared with never smokers and 1.57 (95% CI = 1.03–2.40, P = 0.038) compared with cigarette-only smokers. Among the five components of MetS, the aPORs for elevated WC (2.26, 95% CI = 1.31–3.91, P = 0.003), elevated triglycerides (2.81, 95% CI = 1.90–4.14, P < 0.001), and reduced HDL-cholesterol (2.48, 95% CI = 1.66–3.71, P < 0.001) in dual users were significantly higher than those in never smokers. Also, the aPORs for elevated WC (1.96, 95% CI = 1.19–3.23, P = 0.008) and reduced HDL-cholesterol (1.90, 95% CI = 1.31–2.76, P = 0.001) in dual users were significantly higher than those in cigarette-only smokers (Table 4).

**Table 4:** Multivariate logistic regression analyses for the association of dual use of e-cigarettes and cigarettes with the prevalence of metabolic syndrome and its components.

	Dual users vs. cigarette-only smokers			Dual users vs. never smokers			Cigarette-only smokers vs. never smokers		
	aPOR	95% CI	P-value	aPOR	95% CI	P-value	aPOR	95% CI	P-value
**Elevated WC**									
Model 1	1.72	1.31–2.27	<0.001	2.06	1.55–2.74	<0.001	1.13	0.99–1.29	0.063
Model 2	1.89	1.16–3.09	0.011	2.15	1.24–3.72	0.006	1.04	0.82–1.32	0.757
Model 3	1.96	1.19–3.23	0.008	2.26	1.31–3.91	0.003	1.06	0.83–1.35	0.652
**Elevated BP**									
Model 1	0.72	0.56–0.94	0.016	0.81	0.62–1.07	0.135	1.08	0.95–1.22	0.242
Model 2	0.69	0.49–0.97	0.035	0.63	0.42–0.93	0.021	0.86	0.72–1.04	0.112
Model 3	0.68	0.47–0.98	0.037	0.62	0.41–0.94	0.023	0.87	0.73–1.04	0.134
**Elevated fasting glucose**									
Model 1	1.21	0.89–1.64	0.226	1.62	1.18–2.22	0.003	1.34	1.17–1.52	<0.001
Model 2	1.23	0.80–1.89	0.355	1.35	0.87–2.10	0.186	1.07	0.89–1.29	0.479
Model 3	1.19	0.78–1.82	0.425	1.38	0.89–2.16	0.153	1.1	0.91–1.33	0.333
**Elevated triglycerides**									
Model 1	1.29	0.97–1.71	0.085	2.71	2.03–3.60	<0.001	2.08	1.85–2.33	<0.001
Model 2	1.45	0.99–2.11	0.055	2.75	1.87–4.04	<0.001	1.8	1.53–2.12	<0.001
Model 3	1.44	0.99–2.10	0.058	2.81	1.90–4.14	<0.001	1.83	1.55–2.16	<0.001
**Reduced HDL-cholesterol**									
Model 1	1.54	1.17–2.04	0.002	2.01	1.52–2.67	<0.001	1.28	1.12–1.45	<0.001
Model 2	1.86	1.27–2.72	0.001	2.37	1.59–3.54	<0.001	1.21	1.02–1.44	0.034
Model 3	1.9	1.31–2.76	0.001	2.48	1.66–3.71	<0.001	1.24	1.04–1.49	0.018
**Diagnosis of MetS**									
Model 1	1.33	0.99–1.79	0.057	2.07	1.53–2.80	<0.001	1.46	1.28–1.67	<0.001
Model 2	1.53	1.00–2.33	0.05	2.26	1.44–3.55	<0.001	1.3	1.07–1.58	0.009
Model 3	1.57	1.03–2.40	0.038	2.79	1.72–4.53	<0.001	1.47	1.20–1.82	<0.001

Results are expressed as aPORs (95% CIs) after adjusting for the variables in each model; P-values are calculated using complex samples logistic regression analyses. NOTES: When analyzing the components of MetS, the comorbidity of each item was not adjusted due to the high collinearity between the two variables. Abbreviations: aPOR, multivariate-adjusted prevalence odds ratio; CI, confidence interval; WC, waist circumference; BP, blood pressure; HDL, high-density lipoprotein; MetS, metabolic syndrome.

Model 1: age, educational level, household income, residence location, occupational status, and marital status.

Model 2: Model 1 plus perceived high stress, depressive mood, suicidal thoughts, self-rated health status, alcohol consumption, and body mass index.

Model 3: Model 2 plus comorbidities and family history of diseases.

## Discussion

The primary reasons given by current e-cigarette users for using e-cigarettes were that they helped with smoking cessation. However, most of them (85.3%) simultaneously use conventional cigarette and e-cigarette rather switching completely to e-cigarettes. We demonstrated that dual users were related to greater nicotine dependence, and they had more psychosocial and behavioural risk factors than cigarette-only smokers and never smokers. Dual users were associated with a higher prevalence of MetS and its components in Korean men aged 19 years or older based on a nationally representative survey. Our results showed that most dual users failed to achieve smoking cessation, despite having had more past-year quit attempts and higher intentions to quit than cigarette-only smokers. The average number of CPD was not significantly different between dual users and cigarette-only smokers, but the UC levels were even higher in dual users than in cigarette-only smokers. However, some studies suggested that e-cigarette use may help smokers reduce or quit smoking. The result of internet survey showed that dual use was associated with a significant decrease in CPD after beginning e-cigarette use^11,12^. Martínez *et al*. found that dual users decreased their CPD and cigarette-based nicotine dependence after beginning e-cigarette use but significantly increased their total nicotine intake per day and total nicotine dependence from before to after using e-cigarettes^13^. Piper *et al*. showed that dual users decrease their CPD, but they did not differ from cigarette-only smokers in UC levels, suggesting that they continued to ingest nicotine via e-cigarettes. Dual use is not a sustained pattern for most dual users, but it is more likely to be continued if they are more dependent on e-cigarettes^14^. Hajek *et al*. showed that e-cigarette was more effective for smoking cessation than NRT (18% vs. 9.9%), when both products were accompanied by behaviour support^15^. However, in reality, the e-cigarette has been shown to hardly be of use as a treatment tool for smoking cessation or switching to a less harmful product without the support of healthcare providers. Piper *et al*. showed the result that 48.8% of initial dual users simultaneously used conventional cigarettes and e-cigarettes, and 43.9% reverted to smoking conventional cigarette alone after 1-year follow-up. Only 5.9% switched to e-cigarette completely and 1.4% abstained from both products in a real-world setting^16^. Cigarettes deliver nicotine more efficiently than e-cigarettes; hence, they satisfy the cravings of smokers more quickly and provide a more intense stimulation than e-cigarettes. Therefore, dual users typically prefer cigarettes over e-cigarettes to relieve stress or anxiety and to achieve greater satisfaction for their cravings^17^. Therefore, we suggest that for dual users a more reasonable measure would be to provide evidence-based medication for smoking cessation along with behaviour support by healthcare providers rather than e-cigarettes, until e-cigarettes are proven effective enough. For example, Hajek *et al*. showed that varenicline offered to dual users was likely to promote successful abstinence from both cigarettes and e-cigarettes^18^.

Our results showed that younger age, high educational level, higher incomes, living in urban areas, and professional jobs were more common in dual users that in cigarette-only smokers, as reported previously^19^. Young adults have rapidly adopted e-cigarettes and they are more likely to developing nicotine dependence, e-cigarette manufacturers are targeting them and have developed new-generation e-cigarette devices with high nicotine concentration in e-liquid or with increased voltage for delivering more nicotine steadily. Young adults are also attracted by the novelty and numerous flavour options of e-cigarettes^7^.

Dual users had more psychosocial and behavioural risk factors. First, in this study, dual users had higher proportions of perceived high stress and continuous depressive mood lasting for ≥2 weeks than cigarette-only smokers and never smokers. The proportions of dual users experiencing loneliness, who lived alone, were higher than that of cigarette-only smokers. Despite dual users perceiving their health status as worse than never smokers, dual users with mental health problems have greater nicotine dependence and lower cessation rates than the cigarette-only smokers. Park *et al*. showed that smokers who have high stress levels tend to use both cigarettes and e-cigarettes^20^. E-cigarette use is more prevalent among those with mental illness; this may be due to their perceived benefits, such as ease of smoking cessation or social connectedness. Due to the high rates of smoking in this population, it may be plausible to use e-cigarettes in a harm-reduction model. A new culture centering on e-cigarettes is be formed, which could be appealing to this group as it would allow them to connect with others^21^.

Second, the proportion of high-risk drinking was higher in dual users and cigarette-only smokers than never smokers in this study. Alcohol and tobacco use disorders are closely linked, and some studies have found that e-cigarette use is associated with cigarette smoking, alcohol abuse, and other risky behaviours^22^. Heavy drinkers tend to use e-cigarettes as an additional recreational substance, or could be used in areas where cigarette smoking is prohibited. Because e-cigarette produces a less pungent odour than tobacco smoke, allowing the smoker to feel less uncomfortable in a social setting^22,23^.

Third, dual users ingested more calories per day and had higher proportion of all classes of obesity (I-III) than cigarette-only smokers and never smokers in this study. It is possible that dual users have already accumulated weight through several quit attempts and the failure to quit smoking. Weight gain after smoking cessation remains a barrier to quitting and is a trending theme in the marketing strategies for e-cigarettes. Kim *et al*. showed that post-cessation weight gain is associated with an increased risk of MetS^10^. About 13.5% of adult e-cigarette users reported vaping to lose or control weight^24^. E-cigarettes contain nicotine, as cigarettes do, but the behaviours associated with vaping and the e-cigarette flavours that mimic high calorie/fat foods or beverages can serve as a distraction from or a substitute for eating^24^. However, the effects of e-cigarette and cigarette smoking on weight and metabolic parameters are comparable in animal studies, although this has not yet been studied in humans^25^.

Recent studies have shown that dual users are associated with a higher risk of CVD than cigarette-only smokers^4–6^. The long-term health effects of dual use or e-cigarette use are not well understood yet, but it is well known that several modifiable cardiovascular risk factors are associated with an increased risk of CVD^8,9^. In this study, dual users had more psychosocial and behavioural risk factors mentioned above, and the multivariate logistic regression analyses showed that dual users were associated with a higher prevalence of MetS and its components than cigarette-only smokers and never smokers. Among the five components of MetS, elevated WC, elevated triglycerides, and low HDL-cholesterol were more prevalent in dual users than in never smokers, and elevated WC and low HDL-cholesterol were more prevalent in dual users than cigarette-only smokers, reflecting central MetS. In contrast, the prevalence of elevated BP was significantly lower in dual users than in cigarette-only smokers and never smokers. Smoking has been consistently found to be associated with lower systolic and diastolic BP in Asian populations^26^. The prevalence of elevated fasting glucose levels was not significantly different among dual users, cigarette-only smokers, and never smokers. However, there may be many people who have not yet shown abnormalities in blood sugar and BP levels despite having abdominal obesity. Therefore, early behavioural interventions are critical to prevent MetS.

The strength of this study is that this is the first study to not only compare psychosocial and behavioural risk factors between dual users and cigarette-only smokers in detail but also to confirm that additional e-cigarette use among current smokers was associated with a higher prevalence of MetS and its components after adjustment for multiple potential confounders using data from a large-scale survey with reliable countrywide sampling. It is important for health professionals to have a better understanding of modifiable risk factors among dual users, in addition to providing more active treatments for smoking cessation and intensive lifestyle interventions.

Due to the nature of this study, however, several limitations should be considered when interpreting the results. First, causality, reverse causality, and temporal relationships could not be ascertained based on cross-sectional data. For example, current smokers who had accumulated weight after multiple attempts and failures to quit smoking and had been diagnosed with MetS might prefer starting e-cigarette use to quit smoking. Current smokers with medical and psychiatric problems might start to use e-cigarette to reduce cigarette use or switch to e-cigarettes. The long-term effects of dual use on health outcomes are difficult to study, since e-cigarettes have only been in popular use for a few years, and young adults may not have had enough time to develop clinically significant disease. In this study, the causation between dual use and cardiovascular risk is inconclusive due to potential confounding as well. Further studies using a prospective longitudinal cohort design are needed to verify whether dual use is associated with increased cardiovascular risk. Second, data regarding the type of e-cigarette, the concentration of nicotine in e-cigarettes, the frequency and duration of vaping, and whether the number of CPD decreased after e-cigarette adoption were not available in the KNHANES. Therefore, we could not analyse whether any harmful substances in e-cigarettes are associated with cardiovascular risk factors. It is possible that users of heat-not-burn (HNB) tobacco products were included among those who reported e-cigarette use in this study, but the number of such people is assumed to be very low because these products were introduced in 2017. HNB tobacco products have very different characteristics than e-cigarettes and are more closely related to cigarettes; however, they are commonly confused with e-cigarettes among the Korean users, since the Korean government categorizes HNB tobacco products as “cigarette-type e-cigarettes”, the same classification used for e-cigarettes^27^. Starting in 2019, a survey on e-cigarettes and HNB tobacco products has been added to the KNHANES. Third, one limitation of our study concerns the assessment of nicotine dependence. We could not use the Fagerström test, which is the standard instrument used for assessing nicotine dependence^28^, since it was not conducted in the KNHANES and it could not be applicable for dual users. More reliable and valid measurement tools for assessing e-cigarette dependence in dual users have been developed^29^. Future research using these tools are needed to evaluate nicotine dependence in dual users in Korea. Finally, data regarding socioeconomic status, smoking, and psychosocial and behavioural risk factors were collected via self-reported surveys and, thus, might have been subject to recall bias and underestimation. Furthermore, we may not have fully accounted for potential confounders in the analysis. In conclusion, given that most e-cigarette users are dual users and dual users are more vulnerable to cardiovascular risk factors than cigarette-only smokers and never smokers, more active treatment for smoking cessation and intensive lifestyle interventions for dual users should be considered with priority.

## Methods

### Study population

**Figure 1:**
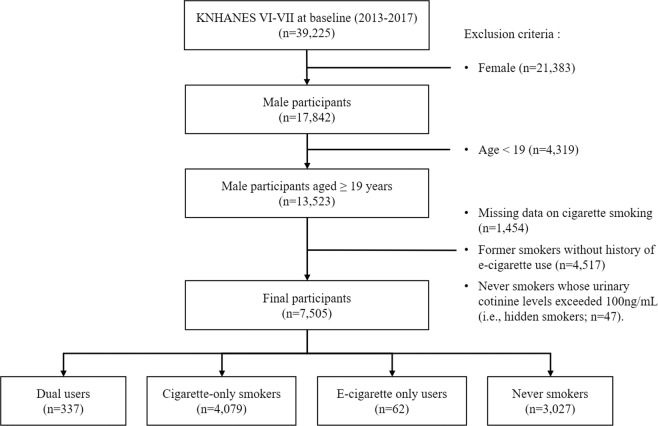
Inclusion and exclusion flow chart.

We analyzed pooled data acquired from the 6^th^ and 7^th^ KNHANES (2013–2017). The KNHANES is a nationally representative cross-sectional survey conducted by the Korea Centers for Disease Control and Prevention and assesses and monitors changes in health risk factors and diseases of Koreans^2^. The initial sample for this study comprised 39,225 participants who had completed the health interview, health behaviour surveys, and health examination surveys (2013–2015, n = 22,948, response rate 78.3%; 2016–2017, n = 16,277, response rate = 76.7%). Female participants (n = 21,383) were excluded due to the low prevalence of smoking and high discrepancy between self-reported and actual smoking status among Korean females^30^. Male participants <19 years of age (n = 4,319) and participants whose documentation showed missing data (n = 1,454) were excluded. Former smokers who did not use e-cigarettes for the past month (n = 4,517) were excluded because confounding effects of smoking cessation cannot be controlled. Never smokers who did not use e-cigarettes for the past month and with UC levels of ≥100 ng/mL (n = 47) were excluded for a more accurate classification of current smokers. Finally, a total of 7,505 participants were included in this study (Fig. 1). We analysed the UC levels for 5,556 participants who took the test for this study after excluding 61 participants who received NRT affecting the UC levels within 5 days.

### Ethical approval and informed consent

The Institutional Review Board (IRB) of Seoul National University Bundang Hospital approved this study (IRB No. X-1909-562-901) and waived the need for obtaining informed consent from the study participants due to the anonymity of the data from the KNHANES database. The study was conducted in accordance with the relevant guidelines and regulations.

### Assessment of smoking status and behaviours

Dual users were defined as those who have smoked more than 100 cigarettes in their lifetime and now smoke every day or some days and have used e-cigarettes for the past month. Cigarette-only smokers were defined as those who have smoked more than 100 cigarettes in their lifetime and now smoke every day or some days but did not use e-cigarettes for the past month. E-cigarette-only users were defined as those who have used e-cigarettes for the past month but currently do not smoke cigarettes. Never smokers were defined as those who have smoked less than 100 cigarettes or have never smoked cigarettes in their lifetime and did not use e-cigarettes for the past month. The frequency of cigarette smoking was categorized into daily or occasional use. The duration of cigarette smoking was calculated as current age minus age of smoking initiation. The pack-years of cigarettes was calculated as the product of the average number of packs of CPD and smoking duration in years. Preparation stages for smoking cessation were categorized into three groups: pre-contemplation stage (no intention to quit smoking), contemplation stage (intention to quit smoking within 6 months), and preparation stage (intention to quit smoking within 1 month). The UC levels were measured using gas chromatography-mass spectrometry and mass spectrometry using a Perkin Elmer Clarus 600 T detector (Perkin Elmer, Finland)^2^.

### Assessment of socioeconomic status, psychosocial and behavioural risk factors

Educational level was divided into elementary school, middle school, high school, and college or higher. Household income quartiles were calculated based on equivalized income (total household income divided by the square root of the number of household members). Residence location, marital status, and occupational status were each categorized into two groups: rural vs. urban area (classified by administrative district), married with spouse present vs. single (including never married, separated, widowed, and divorced), and professional vs. manual labour jobs (including unemployed status).

Psychosocial factors were analysed for variables including perceived high stress, a continuous depressive mood lasting for more than 2 weeks, and suicidal thoughts within the past year. Self-rated health status was measured with the EuroQoL 5-Dimension Questionnaire^31^ and used to assess an individual’s subjective perception of their health status including physical, mental, and social dimensions. Behavioural risk factors were analysed for variables including decreased physical activity, high-risk drinking, higher daily energy intake, and BMI. Physical activity was defined as moderate-intensity activity performed ≥2.5 hours per week, high-intensity activity performed ≥1.25 hours per week, or a combination of moderate-intensity and high-intensity activity. It was assumed that 1 minute of high-intensity activity was equal to 2 minutes of moderate-intensity activity^32^. High-risk drinking was defined as having more than 7 drinks at one time, at least twice a week. Dietary daily energy intake was analysed for daily total caloric intake (1,000 kcal/day). BMI was calculated as weight (kg)/height (m^2^) and categorized into normal (18.5–22.9 kg/m^2^), pre-obese (23–24.9 kg/m^2^), and obese class I-III (≥25 kg/m^2^) according to the Asia-Pacific obesity guidelines^33^. To define comorbidities, participants currently taking anti-hypertensive agents, anti-hyperglycaemic agents (insulin or oral agents), or lipid-lowering agents were considered to have hypertension, diabetes, or hyperlipidaemia, respectively. Participants who reported being diagnosed with myocardial infarction, angina, or stroke were considered to have CVD. Participants whose parents reported being diagnosed with hypertension, diabetes, hyperlipidaemia, or CVD were considered to have a family history of the disease. For the assessment of socioeconomic, psychosocial, and behavioural factors, variables from self-reported questionnaires were used. For the assessment of nutrient status, the usual dietary habits were determined by the 24-hour recall method for assessing dietary intake, and nutrient intake was estimated using the Can-Pro 2.0 nutrient intake assessment software developed by the Korean Nutrition Society^2^.

### Assessment of metabolic syndrome

MetS was defined in accordance with the criteria from National Cholesterol Education Program Adult Treatment Panel III modified for the Asian population^33,34^. The presence of three or more of the following criteria constituted a diagnosis of MetS: (1) WC ≥ 90 cm; (2) systolic BP ≥ 130 mmHg or diastolic BP ≥ 85 mmHg, or taking anti-hypertensive agents; (3) fasting plasma glucose ≥100 mg/dL, or taking anti-hyperglycaemic agents; (4) triglycerides ≥ 150 mg/dL, or taking lipid-lowering agents; and (5) HDL-cholesterol <40 mg/dL, or taking lipid-lowering agents^33,34^. WC was measured at the narrowest point between the upper iliac crest and the lowest rib after normal expiration. Systolic and diastolic BPs were measured by averaging three recordings taken in the morning following at least 10 min of rest in a sitting position using a mercury sphygmomanometer (Baumanometer; Baum, Copiague, NY). Laboratory samples were obtained following an 8-h fast. Blood samples were immediately refrigerated, then transported to the Central Testing Institute in Seoul, Korea and analysed within 24 h. Fasting plasma glucose, triglycerides, and HDL-cholesterol levels were measured using a Hitachi 700–110 chemical analyser (Hitachi, Tokyo, Japan)^2^.

### Statistical analyses

All estimates in the analyses were weighted to represent the Korean population using a complex survey design. Complex samples general linear model and chi-square tests were used to compare continuous and categorical variables, respectively. Continuous variables were expressed as means ± standard error, but the UC levels did not follow a normal distribution so were expressed as median value (interquartile range). Categorical variables were expressed as unweighted numbers (weighted %). Complex samples logistic regression analysis was performed to investigate the association between dual user and the prevalence of MetS and its components using three different models. Model 1 was adjusted for age, educational level, household income, residence location, occupational status, and marital status. Model 2 was adjusted for the variables in Model 1 plus perceived high stress, depressive mood, suicidal thoughts, self-rated health status, alcohol consumption, and BMI. Model 3 was adjusted for the variables in Model 2 plus comorbidities and family history of diseases. Results are expressed as aPOR and 95% CI. When analysing the components of MetS, the comorbidity of each item was not adjusted due to the high collinearity between the two variables. Data analyses were performed using IBM SPSS for Windows ver. 18.0 software (IBM Corp., Armonk, NY, USA). All tests were two-sided, and P < 0.05 was considered statistically significant.

## References

[1] OECD. *OECD Health Statistics: Non-medical determinants of health*, 10.1787/data-00546-en (2019).

[2] Ministry of Health and Welfare; Korea Centers for Disease Control and Prevention. *Korea Health Statistics 2017: Korea National Health and Nutrition Examination Survey (KNHANES)*, https://knhanes.cdc.go.kr/knhanes/index.do (2017).

[3] Maglia M, Caponnetto P, Di Piazza J, La Torre D, Polosa R (2018). Dual use of electronic cigarettes and classic cigarettes: a systematic review. Addict Res Theory.

[4] Osei AD (2019). Association Between E-Cigarette Use and Cardiovascular Disease Among Never and Current Combustible-Cigarette Smokers. Am J Med.

[5] Bhatta DN, Glantz SA (2019). Electronic Cigarette Use and Myocardial Infarction Among Adults in the US Population Assessment of Tobacco and Health. J Am Heart Assoc.

[6] Bhatnagar A, Payne TJ, Robertson RM (2019). Is There A Role for Electronic Cigarettes in Tobacco Cessation?. J Am Heart Assoc.

[7] Glantz SA, Bareham DW (2018). E-Cigarettes: Use, Effects on Smoking, Risks, and Policy Implications. Annu Rev Public Health.

[8] World Health Organization. *Prevention of cardiovascular disease: guidelines for assessment and management of cardiovascular risk*, https://apps.who.int/iris/handle/10665/43685 (2007).

[9] Oh SW (2011). Obesity and metabolic syndrome in Korea. Diabetes Metab J.

[10] Kim K (2019). Weight change after smoking cessation and incident metabolic syndrome in middle-aged Korean men: an observational study. Sci Rep.

[11] Adriaens K, Van Gucht D, Baeyens F (2017). Differences between Dual Users and Switchers Center around Vaping Behavior and Its Experiences Rather than Beliefs and Attitudes. Int J Environ Res Public Health.

[12] Farsalinos KE, Romagna G, Voudris V (2015). Factors associated with dual use of tobacco and electronic cigarettes: A case control study. Int J Drug Policy.

[13] Martinez, U. *et al*. How Does Smoking and Nicotine Dependence Change after Onset of Vaping? A Retrospective Analysis of Dual Users. *Nicotine Tob Res*, 10.1093/ntr/ntz043 (2019). 10.1093/ntr/ntz043 PMC7171272 30883640

[14] Piper ME, Baker TB, Benowitz NL, Kobinsky KH, Jorenby DE (2019). Dual Users Compared to Smokers: Demographics, Dependence, and Biomarkers. Nicotine Tob Res.

[15] Hajek P (2019). A Randomized Trial of E-Cigarettes versus Nicotine-Replacement Therapy. N Engl J Med.

[16] Piper, M. E., Baker, T. B., Benowitz, N. L. & Jorenby, D. E. Changes in Use Patterns OVER ONE YEAR Among Smokers and Dual Users of Combustible and electronic cigarettes. *Nicotine Tob Res*, 10.1093/ntr/ntz065 (2019).

[17] Pokhrel P, Herzog TA, Muranaka N, Regmi S, Fagan P (2015). Contexts of cigarette and e-cigarette use among dual users: a qualitative study. BMC Public Health.

[18] Hajek P (2019). Are ‘dual users’ who smoke and use e-cigarettes interested in using varenicline to stop smoking altogether, and can they benefit from it? A cohort study of UK vapers. BMJ Open.

[19] Jaber RM (2018). Electronic Cigarette Use Prevalence, Associated Factors, and Pattern by Cigarette Smoking Status in the United States From NHANES (National Health and Nutrition Examination Survey) 2013-2014. J Am Heart Assoc.

[20] Park SH, Lee L, Shearston JA, Weitzman M (2017). Patterns of electronic cigarette use and level of psychological distress. Plos One.

[21] Bianco CL (2019). Rates of electronic cigarette use among adults with a chronic mental illness. Addict Behav.

[22] Lee JA, Kim SH, Cho HJ (2016). Electronic cigarette use among Korean adults. Int J Public Health.

[23] Hefner KR, Sollazzo A, Mullaney S, Coker KL, Sofuoglu M (2019). E-cigarettes, alcohol use, and mental health: Use and perceptions of e-cigarettes among college students, by alcohol use and mental health status. Addict Behav.

[24] Morean ME, Wedel AV (2017). Vaping to lose weight: Predictors of adult e-cigarette use for weight loss or control. Addict Behav.

[25] Verhaegen A, Van Gaal L (2017). Do E-cigarettes induce weight changes and increase cardiometabolic risk? A signal for the future. Obes Rev.

[26] Berlin I, Lin S, Lima JA, Bertoni AG (2012). Smoking Status and Metabolic Syndrome in the Multi-Ethnic Study of Atherosclerosis. A cross-sectional study. Tob Induc Dis.

[27] Lee C, Kim S, Cheong YS (2018). Issues of new types of tobacco (e-cigarette and heat-not-burn tobacco): from the perspective of ‘tobacco harm reduction’. J Korean Med Assoc.

[28] Heatherton TF, Kozlowski LT, Frecker RC, Fagerstrom KO (1991). The Fagerstrom Test for Nicotine Dependence: a revision of the Fagerstrom Tolerance Questionnaire. Br J Addict.

[29] Piper, M. E., Baker, T. B., Benowitz, N. L., Smith, S. S. & Jorenby, D. E. E-cigarette Dependence Measures in Dual Users: Reliability and Relations with Dependence Criteria and E-Cigarette Cessation. *Nicotine Tob Res*, 10.1093/ntr/ntz040 (2019). 10.1093/ntr/ntz040 PMC7368344 30874804

[30] Jung-Choi KH, Khang YH, Cho HJ (2012). Hidden female smokers in Asia: a comparison of self-reported with cotinine-verified smoking prevalence rates in representative national data from an Asian population. Tob Control.

[31] Devlin NJ, Brooks R (2017). EQ-5D and the EuroQol Group: Past, Present and Future. Appl Health Econ Health Policy.

[32] Yang YJ (2019). An Overview of Current Physical Activity Recommendations in Primary Care. Korean J Fam Med.

[33] Seo MH (2019). 2018 Korean Society for the Study of Obesity Guideline for the Management of Obesity in Korea. J Obes Metab Syndr.

[34] Expert Panel on Detection, Evaluation, and Treatment of High Blood Cholesterol in Adults (2001). Executive Summary of The Third Report of The National Cholesterol Education Program (NCEP) Expert Panel on Detection, Evaluation, And Treatment of High Blood Cholesterol In Adults (Adult Treatment Panel III). JAMA.

